# 567 Outcomes of an Education Program on the Rehabilitation of Face and Neck Burns

**DOI:** 10.1093/jbcr/irad045.163

**Published:** 2023-05-15

**Authors:** Deborah Knight, Michael Serghiou, Jonathan Niszczak

**Affiliations:** Doctors Hospital of Augusta, Augusta, Georgia; BioMed Sciences, Allentown, Pennsylvania; BioMed Sciences, Philadelphia, Pennsylvania; Doctors Hospital of Augusta, Augusta, Georgia; BioMed Sciences, Allentown, Pennsylvania; BioMed Sciences, Philadelphia, Pennsylvania; Doctors Hospital of Augusta, Augusta, Georgia; BioMed Sciences, Allentown, Pennsylvania; BioMed Sciences, Philadelphia, Pennsylvania

## Abstract

**Introduction:**

Face and neck burns bring unique complications and often require specific training and experience for the burn physical rehabilitation professional. The COVID-19 Pandemic created a need for virtual learning during a time when in-person learning was not possible. While this format can meet some needs, it does not allow for practice and interaction with instructors. There are limited education and training opportunities for burn therapists which include such extensive practical opportunities. This unique didactic training program provided mentorship and tangible skills of a complex and specialized area of burn rehabilitation.

**Methods:**

• Learning Objectives were determined using the Burn Therapist Competency Tool-Version 2

• Developed curriculum for each facial/neck regions as well as various scar management techniques throughout the continuum of care

• Individual modules were developed by Occupational Therapists, Physical Therapist and Speech and Language Pathologist with experience in the field

• The in-person course consisted of 4 hours of lecture and 8 hours of hands-on splinting and scar management training

• Instructors provided interactive and collaborative feedback for every participant Pre & Post testing completed to assess learning

• Feedback from participants was assessed for quality improvement

• Follow up questionnaire was provided to assess implementation into clinical practice and self-perceived level of experience related to rehabilitation of face/neck burns

**Results:**

24 Occupational, Physical and Speech Therapists participated in the 2-day course. Results of the post-test indicate increased knowledge pertaining to the learning objectives. 21 participants remained for the second day of the hands-on splinting and scar management portions and demonstrated learned knowledge of these intervention techniques. Post-course assessments attest to increased comfort with new skills. Participant follow up gaged overall effectiveness of clinical practice improvements.

**Conclusions:**

Participant feedback provided valuable insight into the ongoing need for training and mentoring of burn therapists. This is vital in developing additional training resources, including step-by-step explanation and video content for repetition and learning. Ultimately, implementation of these new skills into each participant’s clinical practice was the desired outcome of this course.

**Applicability of Research to Practice:**

This project highlighted the gap in knowledge for burn therapists when it came to face and neck burns.

